# Optimization of Extraction and Separation Process of Notoginsenoside Fc from *Panax notoginseng* Leaves

**DOI:** 10.3390/molecules28093915

**Published:** 2023-05-05

**Authors:** Zhenghong Guo, Zhonghua Luo, Shao Wu, Chunhong Yang, Ting Xiao, Yuqing Zhao

**Affiliations:** 1 College of Pharmacy, Guizhou University of Traditional Chinese Medicine, Guiyang 550025, China; guo_zhenghong@163.com; 2 Key Laboratory of Natural Medicines of the Changbai Mountain, Ministry of Education, Yanbian University, Yanji 133002, China; 3 China School of Functional Food and Wine, Shenyang Pharmaceutical University, Shenyang 110016, China; zhonghua_2398@163.com (Z.L.); ws_729472403@163.com (S.W.); syych358776815@163.com (C.Y.); 4 The State Key Laboratory of Functions and Applications of Medicinal Plants, The Department of Pharmaceutic Preparation of Chinese Medicine, The High Educational Key Laboratory of Guizhou Province for Natural Medicinal Pharmacology and Druggability, School of Pharmaceutical Sciences, Guizhou Medical University, Guiyang 550025, China; tingjinxiao@126.com

**Keywords:** extraction optimization, *Panax notoginseng* leaves, Notoginsenoside Fc, enrichment

## Abstract

Response surface methodology (RSM) was used to determine the optimal conditions for ultrasound-assisted extraction (UAE) of Notoginsenoside Fc (Fc) from panax notoginseng leaves. The experiment utilized a Box–Behnken design (BBD) and separation conditions were optimized. The optimum extraction conditions were as follows: extraction time = 1.5 h, ethanol concentration = 86%, liquid-to-solid ratio = 19:1. The experimentally obtained values were in accordance with the values predicted by the RSM model. We determined that the RSM model was able to successfully simulate the optimal extraction of Fc from the leaves. Further, Fc was enriched from *Panax notoginseng* through nine macroporous resins, and HPD-100 macroporous resins were selected for preliminary enrichment of Fc due to its economic costs and benefits. Subsequently, octadecyl silane (ODS) column chromatography was used to improve the purity of Fc to over 90% after separation by ODS column chromatography. Fc with a purity greater than 95% can be obtained by recrystallization. This is the first study that has focused on the extraction and enrichment of Fc from *Panax notoginseng* leaves using macroporous resin combined with ODS column chromatography, which provides the possibility for further application of Fc.

## 1. Introduction

*Panax notoginseng* is a famous Chinese herbal medicine and it has been used as a medicinal food and a dietary supplement in many countries [1]. In 1994, it was classified as a dietary supplement by the US Dietary Supplement Health and Education Act. It possesses a lot of biological activity, such as hemostasis, reducing blood fat, antithrombosis, and antineoplastic functions [2]. More than 150 saponins from roots and leaves have been separated [3]. Notoginsenoside Fc (Fc), a novel protopanaxadiol-type (PPD-type) saponin, is isolated from the leaves of *P. notoginseng* [4]. It was reported that it could attenuate HG-induced vascular endothelial cell injury, partly through the upregulation of PPARγ [5]. Fc might reduce renal tubular injury and mitochondrial dysfunction in AKI partly through the regulation of the SIRT3/SOD2 pathway [6]. Fc can accelerate reendothelialization and alleviate excessive neointimal formation following carotid artery injury in diabetic Sprague–Dawley rats in vivo [7]. Fc effectively counteracts platelet aggregation, and the antiplatelet and antithrombotic effects of Fc are carried out through oppression of PLCγ2 and subsequent DAG-PKC-TXA2 and IP3-[Ca^2+^] [8]. Pharmacokinetics, bioavailability, and metabolism of Notoginsenoside Fc in rats by liquid chromatography/electrospray ionization tandem mass spectrometry were also reported [4]. However, no studies have been reported on the extraction and enrichment of Fc from *P. notoginseng* leaves using macroporous resin combined with ODS column chromatography; thus, there is an urgent need to optimize the extracting and enriching conditions.

Many of the bioactivities of *P. notoginseng* are attributed to saponins, which are unique resources for pharmaceutical raw materials, nutraceuticals [9], and food additives [1]. It has been reported that the extraction of saponins is affected by many factors. In the chemical structure of saponins, since aglycones have lipophilicity and sugar chains have strong hydrophilicity, the amphiphilic character gives them the ability to self-micellize, thus increasing their dispersion [10]. It has been reported that the water solubility of saponins depends on the type of saponins [11]. This complicates their extraction process. So far, extraction techniques such as liquid–liquid extraction, solid-phase extraction, aqueous two-phase systems of ionic liquids and salts, and supercritical fluid extraction have been reported for the saponins [12,13]. As a green technology, ultrasonic extraction has been widely used to extract saponins [14]. The cavitation effect of ultrasound can make the cell wall collapse and accelerate the dissolution of intracellular substances [15]. Compared with traditional extraction techniques, ultrasound-assisted extraction (UAE) shortened extraction time, reduced extraction temperature, improved extraction efficiency, saved energy consumption, and did not destroy active ingredients [16].

Current methods for isolating saponins include foam fractionation, various chromatographic materials, preparative HPLC, biotransformation, etc. However, all of these methods have similar drawbacks [17]. The separation of Fc is not easy because it dramatically degrades and is prone to epimerization, particularly at high temperatures [18]. Therefore, it is necessary to optimize the preparation of Fc. Macroporous resin has been widely used for the separation and enrichment of bioactive components from many natural products, such as a green and efficient protocol for industrial-scale preparation of dioscin from Dioscorea nipponica Makino by two-step macroporous resin column chromatography [19]. In addition, macroporous resins are relatively low-cost and easy to regenerate, have high efficiency, and involve less solvent consumption [20].

Response surface methodology (RSM) is widely used in simulation and system dynamics; it is highly credible and able to quickly determine the best experimental conditions for multi-factor systems [21]. It has been used to extract optimization of ginsenoside Rg1 and phenolic content from Korean Red Ginseng and optimization of the enzymatic production of 20 (*S*)-ginsenoside Rg3 from white ginseng extract [22]. The method has the advantages of shorter experiment time, simple use, high precision, and good prediction performance [23]. At present, it is frequently used in chemical, food, and biological optimization processes [24].

The present study focused on optimization of the extraction and enrichment of Fc from *P. notoginseng* leaves, which has not been reported so far. In the extraction process, RSM was used to optimize three independent variables; optimal extraction conditions were confirmed considering Fc content as response. For enrichment of Fc, HPD-100 macroporous resins were used to preliminarily enrich the Fc from *P. notoginseng* leaves, and higher purity of Fc was obtained using combined column chromatography over HPD-100 macroporous resins and ODS. The ODS column can enrich the Fc in a large amount, which is convenient and quick, and improves efficiency.

## 2. Results

### 2.1. Single Factor Experiment

**Figure 1:**
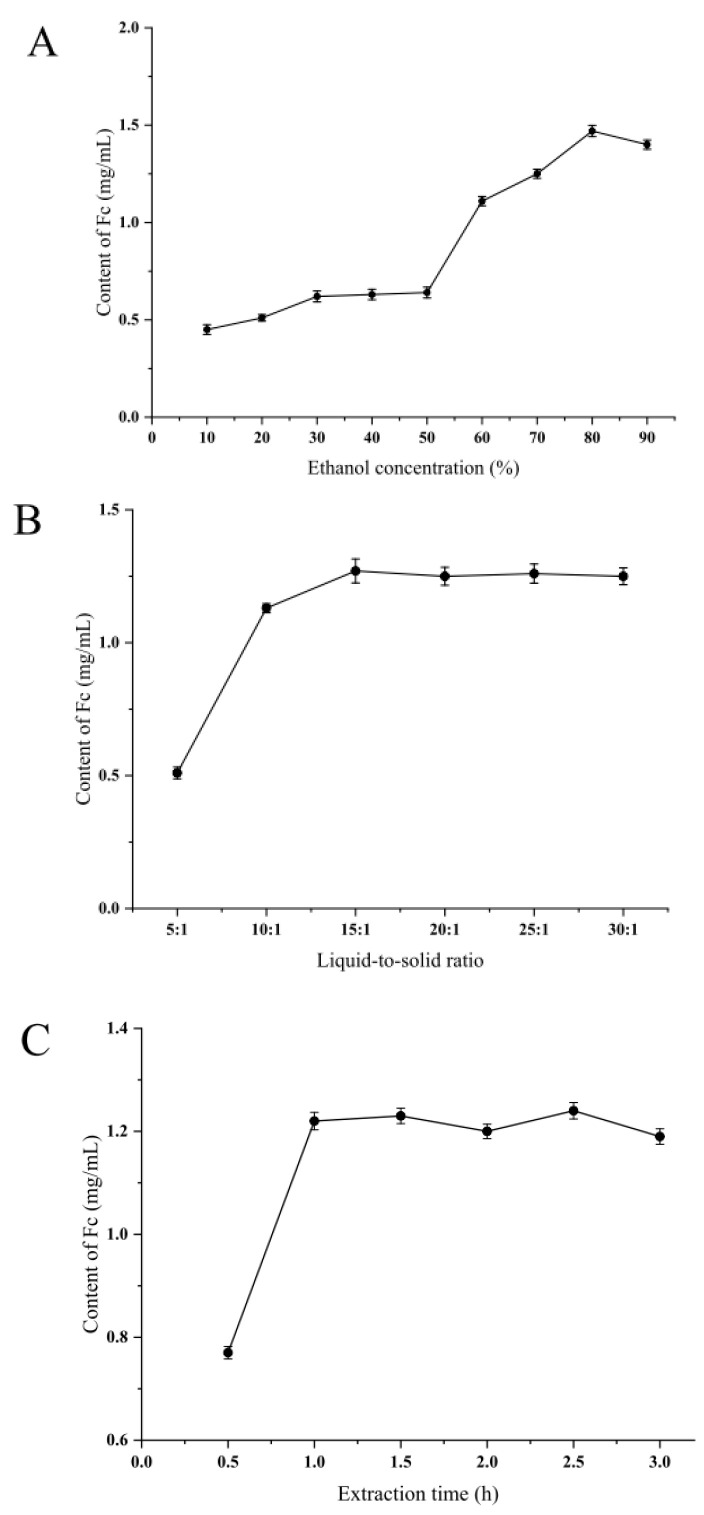
Effect of different extraction factors (**A**) ethanol concentration, %; (**B**) liquid-to-solid ratio, and (**C**) extraction time, h, on the content of Fc.

To obtain a suitable ethanol concentration range, the effect of the ethanol concentration was evaluated at a fixed extraction time (1.5 h) and liquid-to-solid ratio (19:1), as shown in Figure 1A. As the concentration of ethanol increased, the content of Fc was obviously increased until the concentration reached 80%. This phenomenon is most probably due to the high ethanol content leading to high solubility of Fc, which leads to high extraction efficiency. Therefore, 70–90% was chosen as the suitable ethanol concentration range for further optimization.

Solid-to-liquid ratio is a very crucial variable that can significantly affect the extraction efficiency of analyzed compounds in the RSM process. Before building a Box–Behnken design (BBD) model, the effects of extraction parameters on the purity of Fc were determined by a series of preliminary experiments. The parameter liquid-to-solid ratio (5.00–30.00 mL/g) was decided by other fixed variables (extraction time: 1.5 h and ethanol concentration: 86%). The relationship of Fc content is shown in Figure 1B. The concentration of Fc increases with the liquid–solid ratio up to 15 mL/g. When the liquid-to-solid ratio reached 15 mL/g, the increase in liquid-to-solid ratio had little effect on the Fc content. As a result, the ranges of liquid-to-solids (10.00–20.00 mL/g) were chosen for the subsequent experiment. The reason for this phenomenon may be that the liquid-to-solid ratio is relatively small and the Fc is less dissolved, and when the liquid-to-solid ratio is large, the dissolution of the Fc is supersaturated, so it is necessary to select an optimum liquid-to-solid ratio range by a single factor experiment [14].

Under the conditions of ethanol concentration (86%) and liquid-to-solid ratio (19:1 v/m), effects of extraction time (0.5, 1.0, 1.5, 2.0, 2.5, 3.0 h) on the extraction content of Fc were tested. Appropriate extraction time is an important factor for the Fc extraction process. As presented in Figure 1C, like other parameters, the concentration of Fc also increases with an increase in time, and the influence of time on the concentration of Fc gradually decreases when it increases to 1 h. Therefore, 0.5–1 h was selected for subsequent optimization by BBD. The selection of the experimental range for Fc extraction in RSM is not the same, and the plant materials of the conducted research are the key factors [25]. Gong et al. used an extraction time between 7 and 8 h in the extraction of notoginseng from *Panax* [26].

### 2.2. Optimisation of UAE Condition

#### 2.2.1. Analysis of Response Surface Model

The RSM is a statistical comprehensive test technique for dealing with the effects of several variables on a system or structure, that is, the conversion relationship between the input (variable value) and the output (response) of the system or structure. Three factors and three levels of BBD were carried out to optimize the interaction of three independent variables on the content of Fc. The response variable and the independent variables ae presented by the following second-order polynomial equation:

(1) $$\begin{array}{l} Y=1.63+0.0906X1+0.090X2+0.24X3+0.047X1X2+0.053X1X3+0.11X2X3\\ -0.13X1^{2}-0.13X2^{2}-0.30X3^{2} \end{array}$$

In the equation, X_1_, X_2_, X_3_ represent the ethanol concentration, liquid-to-solid ratio, and extraction time, respectively. The positive and negative values of the coefficients denote the degree and direction of the influence of the independent variable and the interactive terms of each factor on the Y value, and Y is the content of Fc. It can be seen from the formula that the extraction time (X_3_) has the greatest influence on the Fc content.

The analysis of variance (ANOVA) test (F-test) was used to check the statistical significance of the second-order polynomial equation (Table 1) [27]. Principally, the *p*-value was <0.05, suggesting the significance of the model terms. The *p* values of ethanol concentration (X_1_), solid-liquid ratio (X_2_), and extraction time (X_3_) were all less than 0.01, which was extremely significant, indicating that the selected factors had significant impacts on the response value. The interaction coefficients of liquid-to-solid ratio (X_2_) and extraction time (X_3_) were significant due to the corresponding *p*-value (*p* < 0.05). The experimental value R^2^ = 98.68%, greater than 90%. The higher R^2^ not only represents the majority of the variables that can be explained by the model, but also indicates that the experimental data are very consistent with the second-order polynomial equation [28]. The result of Adj R^2^ value should be close to R^2^ in a good experimental design [29]. In this experiment, Adj R^2^ = 96.97%, which differed from R^2^ by 1.71%, demonstrating that the experimental model had a high degree of fit. The model F-value, 57.94, implied that the model was significant. The model’s *p*-value was less than 0.01, implying that the model was significant. The lack of fit was an important data point used to evaluate the reliability of the equation [30]. The lack of fit with *p*-value was 0.1178, which was greater than 0.05, and insignificant. It indicated that the selected model was sufficiently accurate in predicting the relevant response. The coefficient of variation (CV) disclosed the credibility and precision of the experiment [31]. It could be seen that the CV in Table 2 was 3.56%, less than 10, which indicated the reproducibility of the model. In addition, the PRESS was 0.20, which was within the acceptable range [32].

**Figure 2:**
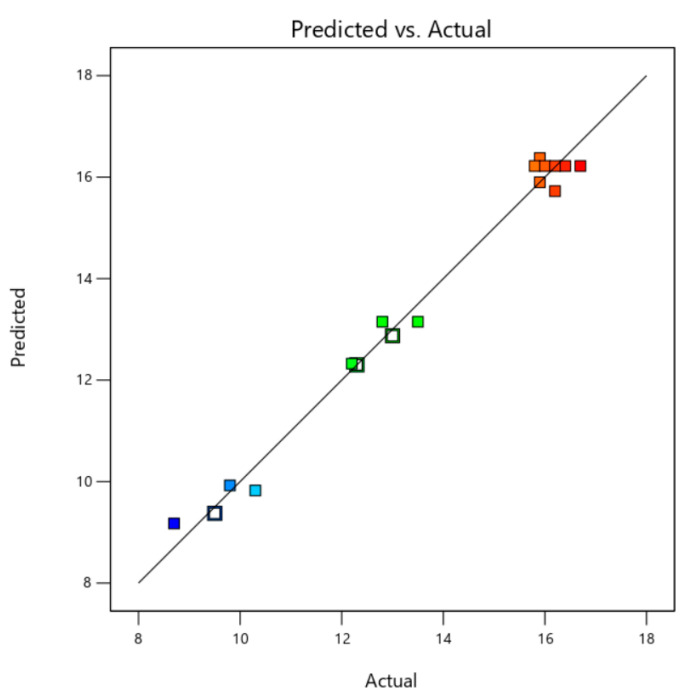
The comparison between the predicted and measured values of the content of Fc.

The above important parameters proved the suitability of the model. In addition, the predicted value of the model and the actual value of the experiment were fitted almost in a straight line, which also certified that the second-order polynomial regression model was consistent with the experimental results (Figure 2). Therefore, the models in the experiment can identify the operating conditions for selective extraction of Fc.

#### 2.2.2. Interactions of Different Experimental Factors on the Effects of Response Variables

**Figure 3:**
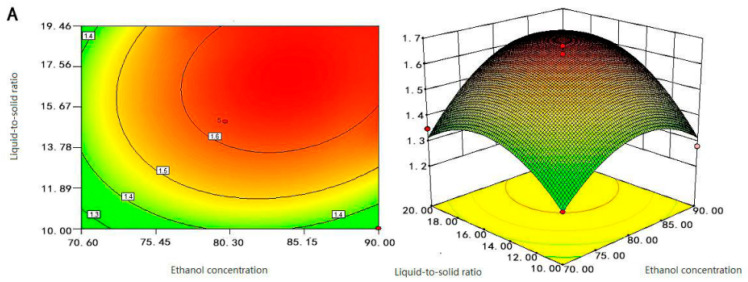

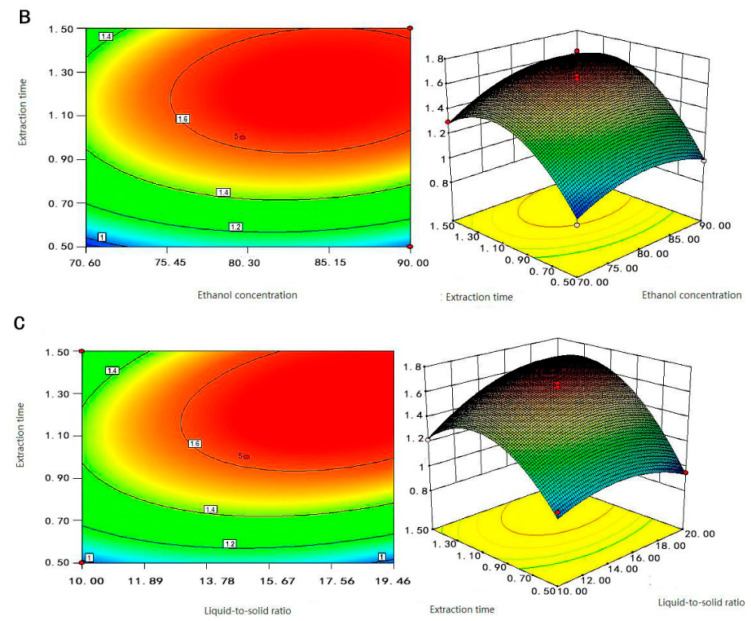
Response surface and contour plots of (**A**) liquid-to-solid ratio and ethanol concentration, (**B**) extraction time and ethanol concentration, and (**C**) extraction time and liquid-to-solid ratio on the content of Fc.

As a classical modeling method, the objective of RSM is to simultaneously optimize the levels of these variables to attain the best system performance, which has advantages over classical one-variable-at-a-time optimization, such as the generation of large amounts of information from a small number of experiments and the possibility of evaluating the interaction effect between the variables on the response. Contour maps of different shapes provide different information for the interaction between variables [33]. The experimental results are presented in Figure 3 as the response surface plot and contour plot. The results revealed the nature of the fitted surface as a maximum, a minimum, or a ”saddle point” [34]. They revealed the effect of two experimental variables on Fc content, while the third variable remained unchanged, examining the effect of interaction terms on Fc content. The steeper the slope of the response surface was, the flatter the contour line was, and the more dense the arrangement was, indicating that the Fc content was sensitive to change in various factors, and the effect was significant. If the shape of the contour plot has an elliptical shape, the interaction between the corresponding factors is significant. In addition, the circular shape of the contour plot indicated that the corresponding factors are negligible [27].

As shown in Figure 3A, when the ethanol concentration was between 75–85%, and the liquid-to-solid ratio was between 14–20 mL/g, the Fc content obtained was the largest. After that, the content of Fc decreased gradually regardless of the increasing or decreasing of ethanol concentration and liquid-to-solid ratio. The contour of the liquid-to-solid ratio axis was denser than that of the ethanol concentration axis, indicating that the effect of liquid-to-solid ratio on Fc content was greater than that of ethanol concentration. It was reported that the liquid-to-solid ratio was relatively small and the viscosity of the solution was larger; there was a low molecular diffusion rate and small extraction rate, and as the liquid-to-solid ratio increased, the velocity of molecular diffusion became faster [35].

Just as the analysis in Figure 3A,B suggests, when the ethanol concentration was between 75–85%, and the extraction time was between 1.1–1.5 h, the Fc content of the extraction was the largest. The effect of extraction time on the content of Fc was greater than the concentration of ethanol. If the extraction time was too short and the Fc was not completely extracted, and if the extraction time was too long, the other alcohol-soluble impurities would also be dissolved and extracted, which hindered the dissolution of Fc. Figure 3C shows that when the liquid-to-solid ratio was between 14–20 mL/g, and the extraction time was between 1.1–1.5 h, the content of Fc was the largest. The steepness of the extraction time was greater than the liquid-to-solid ratio which suggested that the extraction time was the leading factor. These findings agree with Djamila Belhachat et al. [30]. Therefore, we concluded that the extraction time was the main determinant parameter to the Fc extraction process, followed by the liquid-to-solid ratio, and followed by the concentration of ethanol.

In the comprehensive analysis of Figure 3, the response surface maps of the liquid-to-solid ratio and extraction time changed steeply, its contours were sparse and elliptical, and the ellipse radians was large. The results showed that the interaction between liquid-to-solid ratio and extraction time was significant, and they both significantly affected the content of Fc. The response surface of ethanol time and extraction concentration changed more gently; its contours were dense and slightly elliptical, which indicated that the interaction between ethanol concentration and extraction time was not significant and they had less effect on the content of Fc. The response surface of ethanol concentration and liquid-to-solid ratio were relatively flat, the contours were dense and round, and the interaction between ethanol concentration and liquid-to-solid ratio was not significant. Therefore, the influence of the interaction term on the content of Fc was X_2_X_3_ > X_1_X_3_ > X_1_X_2_; this was consistent with the analysis of variance in Table 1. Therefore, RSM could be effectively carried to Fc extraction by optimizing factors such as ethanol concentration, solid-liquid ratio, and extraction time of samples.

#### 2.2.3. Determination and Validation of Optimized UAE Condition

Based on Figure 3, the optimal extraction conditions for Fc were obtained at an ultrasonic time of 1.29 h, ethanol concentration of 85.73%, and liquid-to-solid ratio of 18.52:1 mL/g. Considering the convenience and feasibility of the experimental operation and the accuracy of the equipment and instruments, the optimal experimental conditions were adjusted to the ethanol concentration of 86%, the liquid-to-solid ratio of 19:1 mL/g, and the extraction time of 1.5 h. After three rounds of extraction under these optimal conditions, the theoretical optimum value of Fc content was 17.30 mg/g, which was not significantly different from the predicted value of 17.50 mg/g, and the RSD% was 1.78%, which expressed that the model fitted well with the actual situation, and the model was reliable.

### 2.3. Optimum Macroporous Resin

Macroporous resin has been widely used in plant enrichment and purification of active compounds, with high purity, easy operation, and reusability [36,37]. Meanwhile, many studies have shown that the extraction of Notoginsenoside can involve different concentrations of ethanol, including 70%, 75%, 80%, 85% ethanol, etc. [25,26].

**Figure 4:**
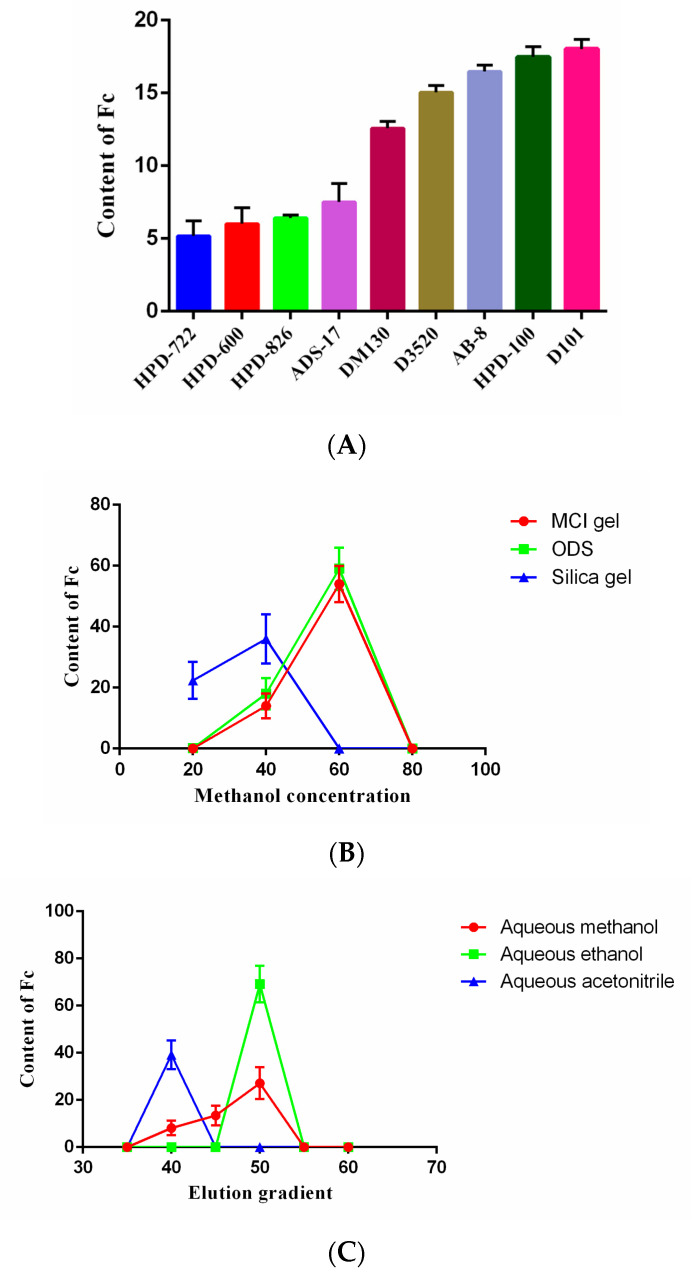
Separation process of Notoginsenoside Fc. Effect of different types of macroporous resins on the enrichment of Notoginsenoside Fc; (**A**) Content of Fc in eluents with different fillers and gradients; (**B**) The content of Fc in different elution gradients of different elution solvents (**C**).

The ability of different types of resins to adsorb and desorb Fc is related to their chemical and physical properties such as functional group, polarity, and surface area. It can be seen in Figure 4A that the non-polar resins and weakly polar resins had a good enrichment effect on Fc compared to other types of resins. The best enrichment effect of Fc was found for the D101 resins, probably due to the fact that the resin of D101 had higher average pore diameter (100–110 nm) than HPD-100 (85–90 nm). The enrichment effect of weakly polar AB-8 resins on Fc was second only to HPD-100 resins; the enrichment effect of non-polar D3520 resins was also lower than HPD-100 resins. All these results indicated that polarity and surface area of the resins were both important for the enrichment of Fc. In terms of economic costs and benefits, HPD-100 macroporous resins were selected as the preliminary enrichment resin. Moreover, 70% aqueous ethanol was applied as a reference solvent for elution of Fc from macroporous resin [37]. These results illustrated that HPD-100 resins could effectively adsorb the Fc, which could be obtained with ethanol after eluting the impurities with deionized water. In addition, the Fc prepared in this study was cost-effective and environmentally friendly, with no waste of organic solvents, use of expensive materials, or need for special instruments.

### 2.4. Determination of Secondary Separation Column Chromatography

The comparison of the three fillers is presented in Figure 4B. Fc was found in the 60% aqueous methanol eluents of Middle Chromatogtam Isolated (MCI) gel and ODS, and in the 20–40% aqueous methanol eluates of silica gel. However, the content of Fc was highest in the 60% aqueous methanol eluates of ODS; therefore, the ODS was used as the secondary enrichment filler. Purification and enrichment of saponin using ODS column chromatography has been widely investigated [38]. ODS, octadecylsilane, is a silica-based C18 filler and belongs to reversed-phase chromatography. It is suitable for the separation of compounds with moderately large polarities. There are many successful separation and purification examples, such as the performance and separation characteristics on the column packed with MCI gel, D101 macroporous resin and ODS for the enrichment and separation of oleanane-type triterpenoid saponins with 3, 28-O-bidesmosides (OTSB), and oleanane-type triterpenoid saponins with 3-O-monoglucosides (OTSM) from then-BuOH-soluble portion of Gypsophila pacifica Kom [31]. In summary, the separation of Fc from *P. notoginseng* leaves was achieved for the first time using combined column chromatography over HPD-100 resins and ODS.

### 2.5. Effect of Different Elution Solvents on ODS

**Figure 5:**
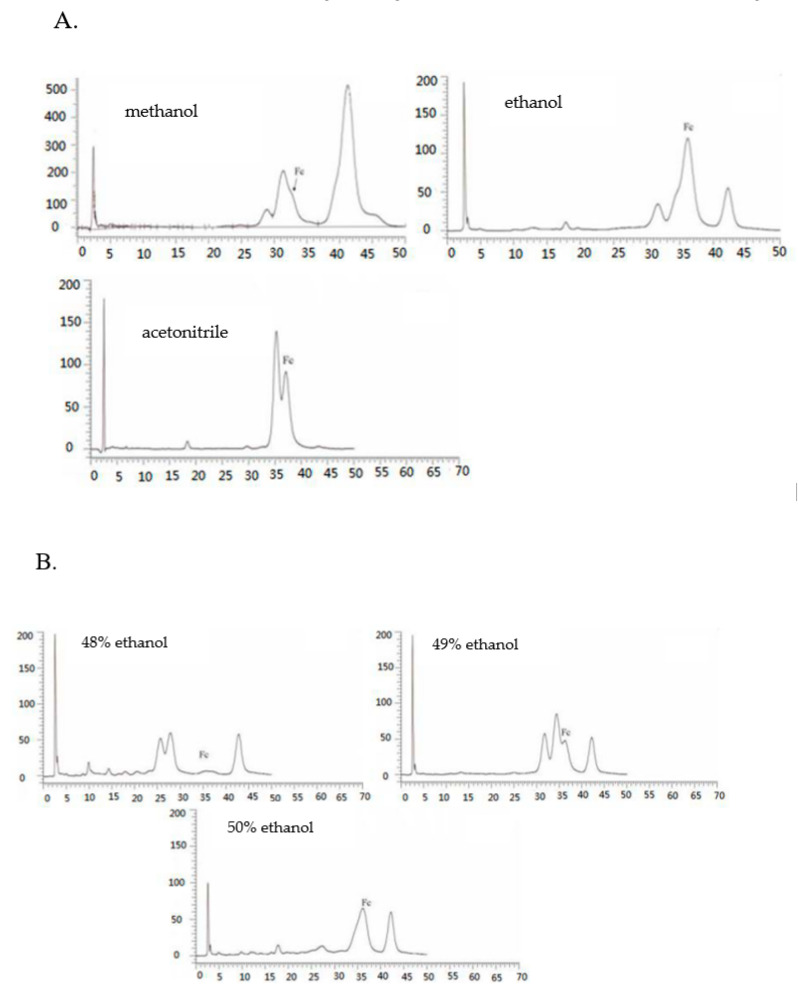

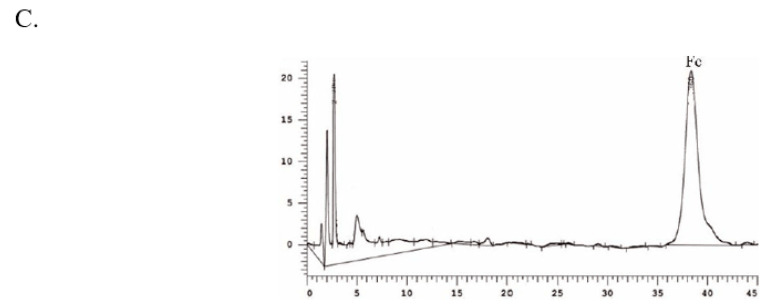
Determination of secondary separation column chromatography. High performance liquid chromatogram of methanol elution, ethanol elution, and acetonitrile elution fraction on ODS (**A**); High performance liquid chromatogram of 48%, 49%, 50% ethanol eluent on ODS (**B**); HPLC chromatograms of the extracts after separation using the macroporous gel and ODS (**C**).

Figure 4C shows that among all the tested solvents, aqueous ethanol possessed the best enrichment effect on Fc, and the elution of Fc using aqueous methanol and aqueous ethanol was found to be in the range of 45−50%, while the aqueous acetonitrile was found in the 40% eluent. It can be seen from Figure 5A that the purity of Fc was low in the aqueous methanol elution fraction and it was difficult to separate. The purity of Fc in the ethanol elution fraction was higher while it was relatively low in acetonitrile elution. The results also showed that the best separation effect of Fc was obtained from aqueous ethanol fraction. The selection agreed with a previous study for the preparative separation of minor saponins from *P. notoginseng* leaves using aqueous ethanol (*v/v*) [30]. Moreover, the cost of ethanol is also low; it is easy to recycle, economical, and environmentally friendly.

### 2.6. The Optimal Separation Process and HPLC Analysis of Notoginsenoside Fc in P. notoginseng Leaves

Based on the results and evidence in our study, the separation of Fc fraction enriched by the optimized HPD-100 macroporous resins was revealed as follows. The appropriate elution gradient is essential for the acquisition of target compounds. In addition, 49% and 50% of the aqueous ethanol eluent contained a large amount of Fc (Figure 5B), but the purity of Fc was 28% and 52%, respectively. The impurities with similar polarity cannot be removed, and the effect of purification was not achieved. Referring to Figure 3C, in the fractions of aqueous acetonitrile, impurities similar to the polarity of Fc can be separated. In a sum, the purified Fc by ODS chromatography was eluted 5 bed volume (BV) with 48% aqueous ethanol; the impurities near the Fc were removed. Next, the 35% aqueous acetonitrile solution with high polarity and good separation were applied, and the impurities were removed with 5 BV, then eluted with 40% aqueous acetonitrile solution. The HPLC chromatogram of the Fc obtained from the ODS chromatography can be seen in Figure 5C, which shows that after the combination of aqueous ethanol and aqueous acetonitrile, the purity of Fc surpassed 90%. A higher purity (>95%) of Fc can be obtained by a single crystallization operation. This proves that the elution process was reasonable.

In this paper, the enrichment process of Fc from *P. notoginseng* leaves was studied for the first time. Firstly, the enrichment of Notoginsenoside in *P. notoginseng* leaves was followed by the enrichment of Fc from Notoginsenoside; this was divided into two steps. Through experiments, the two-step processes of enriching packing, solvent, and the gradient of the selected solvent were determined. The instruments used in this process were all conventional instruments, while no precision instruments such as preparative high performance liquid chromatography were used. In our study, the open ODS column can be used to enrich a large amount of Fc, which was convenient and fast and improved the efficiency; it was also suitable for the preparation of a large number of samples.

## 3. Materials and Methods

### 3.1. Materials

The *P. notoginseng* leaves was purchased from Wenshan Nanyao Sanqi Industry Co., Ltd. (Yunnan, China). Product batch number: PN20160320. The reference substance was Fc (self-made in the laboratory, >99% by HPLC). Methanol (chromatography and analytical grade) and ethanol (analytical grade) were purchased from Concord Chemical Reagents Co. (Tian-jin, China).

### 3.2. Extraction Process

The leaves were dried and pulverized, and passed through a 100-mesh sieve. The powdered samples (2.0 g) were mixed with different concentrations of ethanol (70–90%) using different solid-to-solvent ratios (1:10–1:20). These samples were extracted in a sonication water bath (KQ2200B, KunshanUltrasonic Equipment Co., Ltd., Kunshan, China) for different time periods (0.5–1.5 h). Then, the sample solution was taken out and centrifuged, and the obtained supernatant was decompressed to recover ethanol.

### 3.3. Experimental Design and Statistical Model

The RSM was used to optimize the ultrasonic extraction of Fc to decide the best extraction process. The experiment was based on the Box–Benhnken design (BBD) method. The three variables of ethanol concentrations, liquid-to-solid ratios, and extraction times were investigated. The content of Fc in the sample was determined as the response variable. There were 17 groups of experiments (Table 2), 12 of which were analytical experiments and 5 of which were central experiments. Statistical calculations of the variables were coded by the equation:

(2) $$Y=A_{0}+\sum_{i=1}^{3}A_{i}X_{i}+\sum_{i=1}^{3}A_{ii}X_{i}{}^{2}+\sum_{i=1}^{2}\sum_{j=i+1}^{3}A_{ij}X_{i}X_{j}$$

*Y* represents the response variable; *A*_0_ represents the intercept constant, *A_i_*; *A_ii_* and *A_ij_* are the regression coefficients for the linear, the quadratic, and the interaction, respectively. *X_i_* and *X_j_* are coded values of the independent variables.

Multivariate quadratic regression equations were used to approximate the relationship between factors and response values, and regression equations were analyzed to find optimal process parameters.

### 3.4. HPLC Analysis

L-2200 HPLC (Hitachi, Tokyo, Japan) was employed to determine the Fc content, equipped with Promosil C18 chromatographic column (250 mm × 4.6 mm, 5 μm). The mobile phase A was methanol, and B was water. A mixture of 65% A and 35% B were used for isocratic elution at 1.0 mL/min at 35 °C and 203 nm. The injection volume was 10 μL. The quantification was conducted using the Fc standard solution. Fc had a good linear relationship when the concentration was 0.005−1.000 mg/mL, and the calibration curve was linear with R^2^ = 0.9996.

### 3.5. Seperation Process of Notoginsenoside Fc

#### 3.5.1. Selection of Macroporous Resins for Preliminary Separation of Fc

Macroporous resins, including AB-8, D3520, ADS-17, HPD100, HPD826, D101, HPD600, DM130, and HPD722, were purchased from Cangzhou Baoen Co. Ltd. (Cangzhou, China) (Table 3). According to the specification, the macroporous resins were pretreated and washed with deionized water until neutral.

The 9 parts of the powder of leaves which were screened through a 40-mesh sieve were accurately weighed, at 2.00 g each. The samples were respectively added with 38 mL 86% ethanol solution and ultrasonically extracted for 1.5 h, and then centrifuged. The supernatant was concentrated to eliminate alcohol, and loaded onto the 9 resin columns. After the sample solution was adsorbed, the column was sequentially eluted with deionized water and 70% ethanol solution. Then, 50 mL of the eluate was collected as a fraction. The fractions of ethanol were concentrated and analyzed by HPLC. The preliminary separation of crude extracts from different types of macroporous resins was investigated.

#### 3.5.2. Selection of Secondary Separation Column Chromatography

The sample of Fc separated by HPD-100 was dissolved in methanol for 10–15 mg/mL as a sample solution, and loaded onto Silica gel, MCI gel, and ODS column chromatography, respectively. Then, the column was sequentially eluted with 20%, 40%, 60%, and 80% methanol, and each gradient was eluted with 3 BV; the eluent was recovered and analyzed by HPLC.

#### 3.5.3. Effect of Different Elution Solvents on ODS

The Fc sample separated by the HPD-100 was dissolved in an appropriate amount of methanol solution and loaded onto ODS column. Elution was carried out with aqueous methanol, aqueous ethanol and aqueous acetonitrile, respectively. The elution gradients were 35%, 40%, 45%, 50%, 55%, and 60%. The eluents of each elution gradient were collected and concentrated to dryness. The purity of Fc in the fraction was determined by HPLC to investigate the effect of different elution solvents.

#### 3.5.4. Separation Process

The Fc sample separated by the HPD-100 was loaded onto an ODS column. The column was eluted with 45%, 46%, 47%, 48%, 49%, and 50% aqueous ethanol in sequence, and eluted with 5 BV to collect the elution of each gradient. The effect of different gradient ethanol elutions on Fc purity was investigated by HPLC. Then, the column was washed with 35% acetonitrile to remove impurities, and the column was further eluted with 40% acetonitrile. The Fc in 40% aqueous acetonitrile eluate was determined by HPLC.

### 3.6. Crystallization of Notoginsenoside Fc

The 2.0 g Fc sample was prepared to a saturated solution by 95% ethanol at 80°C. Then, the saturated solution was transferred into a 500 mL Erlenmeyer flask at a crystallization temperature of 25 °C. It was crystallized for 5 days and the crystals of Fc was separated from the liquid. The crystalline surface was washed with a small amount of the cold 95% ethanol solution for several times, then dried at 80 °C.

## 4. Conclusions

**Figure 6:**
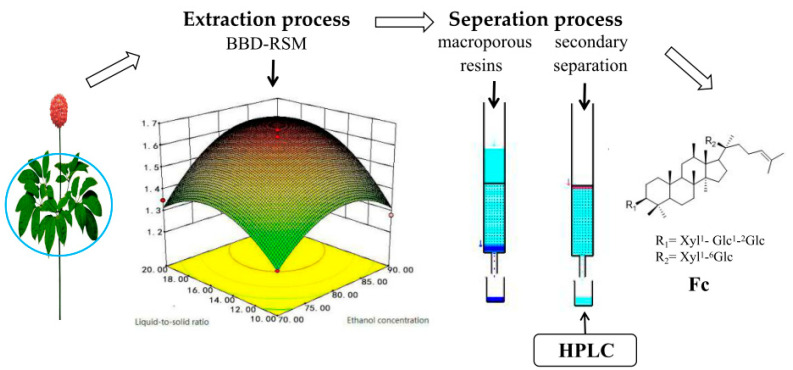
The process diagram of extraction and separation process of Notoginsenoside Fc from *P. notoginseng* leaves.

In our study, the ultrasonic extraction process of Fc from *P. notoginseng* leaves was optimized by using RSM. Due to the single factor test, BBD was designed to evaluate and optimize extraction variables (extraction time, ethanol concentration and liquid-to-solid ratio) to improve the content of Fc. The optimal extraction conditions of Fc were an ethanol concentration of 86%, a liquid-to-solid ratio of 19:1 mL/g, and extraction time of 1.5 h, which resulted in a theoretical optimal value of Fc content of 17.30 mg/g. The separation of Fc was examined using a series of fillers and several series eluents. The optimum macroporous resin for preliminary separation of Fc was HPD-100 resins, using deionized water to remove impurities, and the optimum gradient for eluting the Fc sample solution was 70% ethanol. After the prefractionation of HPD-100 resins, the purity of Fc was greater than 30%. During further enrichment treatment using ODS column chromatography, after elution of 5 BV with 48% ethanol water and then excluding impurity volume of 35% acetonitrile with water, 5 BV; a higher purity Fc (>90%) can be obtained. This was done by eluting with 40% aqueous acetonitrile fraction. Fc with a purity greater than 95% can be obtained by single crystallization operation. The yield of Fc is 0.98% of the extraction and separation process. The proposed method is convenient and quick, and improves efficiency. The process diagram of extraction and separation process of Notoginsenoside Fc from *P. notoginseng* leaves is in Figure 6.

## Figures and Tables

**Table 1:** Analysis of variance, regression coefficients, and quadratic equations of the Fc content response surface models.

Source	Sum of Squares	Df	Mean Square	F Value	*p*-Value		
model	1.22	9	0.14	57.94	<0.0001	Significant	**
X_1_	0.065	1	0.065	27.62	0.0012		**
X_2_	0.065	1	0.065	27.2	0.0012		**
X_3_	0.45	1	0.45	192.37	<0.0001		**
X_1_X_2_	9.025 × 10^−3^	1	9.025 × 10^−3^	3.85	0.0906		
X_1_X_3_	0.011	1	0.011	4.70	0.0668		
X_2_X_3_	0.051	1	0.076	21.58	0.0024		*
${X_{1}}^{2}$	0.076	1	0.076	32.35	0.0007		**
${X_{2}}^{2}$	0.070	1	0.070	29.99	0.0009		**
${X_{3}}^{2}$	0.38	1	0.38	160.74	<0.0001		**
Residual	0.016	7	2.346 × 10^−3^				
Lack of Fit	0.012	3	4.033 × 10^−3^	3.73	0.1178	Not Significant	
Pure Error	4.320 × 10^−3^	4	1.080 × 10^−3^				
Cor Total	1.24	16					
Std.Dev	0.048						
Mean	1.36	R2	0.9868				
C.V.%	3.56	Adj R2	0.9697				
PRESS	0.20						

** < 0.01, * < 0.05.

**Table 2:** Response surface methodology of a three-variable three-level BBD and corresponding response values.

Run	Ethanol Concentration (%)—X_1_	Solid to Solvent Ratio (ml/g)—X_2_	Extraction Time (h)–X_3_	Fc Content (mg/g)
1	90	20	1	15.9
2	80	15	1	16.7
3	80	15	1	16.4
4	90	15	1.5	16.2
5	70	10	1	12.3
6	90	10	1	12.8
7	80	10	0.5	10.3
8	80	15	1	16
9	70	15	0.5	8.7
10	90	15	0.5	9.8
11	80	20	0.5	9.5
12	80	10	1.5	12.2
13	80	15	1	16.2
14	70	15	1.5	13
15	80	15	1	15.8
16	80	20	1.5	15.9
17	70	20	1	13.5

**Table 3:** Physical and chemical properties of the tested macroporous resins.

Macroporous Resin	Polarity	Surface Area (m^2^/g)	Average Pore Diameter (nm)	Particle Diameter (mm)
AB-8	Weak-polar	480–520	130–140	0.3–1.25
D101	Non-polar	400–600	100–110	0.3–1.25
HPD100	Non-polar	650–700	85–90	0.3–1.2
HPD600	polar	550–600	80	0.3–1.2
D3520	Non-polar	480–520	85–90	0.3–1.25
HPD826	Weak-polar	500–600	9–10	0.3–1.25
ADS-17	Weak-polar	90–150	25–30	0.3–1.25
HPD722	Weak-polar	485–530	13.0–14.0	0.30–1.25
DM130	Weak-polar	500–550	9.0–10.0	0.25–0.84

## Data Availability

The data that support the findings of this study are available from the corresponding author upon reasonable request.

## Sample Availability

Samples of the *P. notoginseng* Leaves are available from the authors.
